# Global trend and predictors of non-labelled sacubitril–valsartan dosing: results from IKNOW-HF survey

**DOI:** 10.1093/eschf/xvag162

**Published:** 2026-06-06

**Authors:** Israa Fadhil Yaseen, Hasan Ali Farhan, Justin Paul Gnanaraj, Hidehira Fukaya, Hawani Sasmaya Prameswari, Clara Saldarriaga, Tuğba Kemaloğlu Öz, Somaya Aref Elhout, Pablo Díez-Villanueva, Maria Rosa Costanzo, Mustafa Toma, Nafisa Omar Elsammani Elsheikh Ibrahim, Zainab Atiyah Dakhil, Nathan Mewton, Wilfried Mullens, IKNOW-HF investigators Group, Alexander Lyon, Karen Sliwa, Dania Mohty, Peter P Rainer, Yasmin Rustamova, Mariya Tokmakova, Anders Barasa, Julie K K Vishram-Nielsen, Amr Abdin, Peggy Kostakou, Novi Yanti Sari, Zainab Mustafa Mahdi, Marta Zaleska Kociecka, Rasha Kaddoura, Laura Antohi, Dania Mohty, Mirvat Alasnag, Marija Zdravkovic, Goran Loncar, Ivan Milinković, Anja Zupan Mežnar, Karen Sliwa, Carmen Basic, David Niederseer, Federica Guidetti, Alexander Lyon, Priya Reehal, Ilya Giverts, Oumol Khairy El Moctar El Houssein

**Affiliations:** Baghdad Heart Center, Baghdad Teaching Hospital, Baghdad Medical City, Baghdad, Iraq; Baghdad Heart Center, Baghdad Teaching Hospital, Baghdad Medical City, Baghdad, Iraq; Iraqi Board for Medical Specializations, Scientific Council of Cardiology, Baghdad, Iraq; University of Baghdad, College of Medicine, Baghdad, Iraq; Institute of Cardiology, Madras Medical College, Parktown, Chennai, Tamil Nadu 600003, India; Department of Cardiovascular Medicine, Kitasato University School of Medicine, Kanagawa, Japan; Cardiovascular Medicine Department, Hasan Sadikin General Hospital, Faculty of Medicine, Padjadjaran University, Bandung, West Java, Indonesia; Cardio VID Clinic, Medellin, Colombia; Flinders Medical School, Flinders University, Adelaide, SA, Australia; School of Medicine, University of Notre Dame Australia, Fremantle, WA, Australia; Cardiology Department, Hollywood Private Hospital, Nedlands, WA, Australia; Cardiology Department, Al-Shifaa Medical Complex, Gaza, Palestine; Cardiology Department, Al-Aqsa Martyrs Hospital, Gaza, Palestine; Department of Cardiology, Hospital Universitario de la Princesa, Madrid, Spain; Midwest Cardiovascular Institute, Naperville, IL, USA; Division of Cardiology, University of British Columbia, Vancouver, British Columbia, Canada; Department of Internal Medicine, Faculty of Medicine, Al Neelain University, Khartoum, Sudan; Cardiology Department, Ibn Al-Bitar Cardiac Centre, Baghdad, Iraq; Al-Kindy College of Medicine, University of Baghdad, Baghdad, Iraq; Institut de Cardiologie des Hospices Civils de Lyon, Université Claude Bernard Lyon, Lyon, France; Department of Cardiology, Ziekenhuis Oost-Limburg, Genk, Belgium; Hasselt University, Biomedical Research Institute, Faculty of Medicine and Life Sciences, LCRC, Diepenbeek, Hasselt, Belgium

**Keywords:** Angiotensin receptor–neprilysin inhibitor, Heart failure, Guideline-directed medical therapy

## Abstract

**Background and Aims:**

Optimizing guideline-directed medical therapy (GDMT) remains vital for heart failure (HF) management. However, non-labelled dosing of sacubitril–valsartan is increasingly reported.

This global survey characterized real-world sacubitril–valsartan prescribing patterns and evaluated pharmacist involvement in HF teams.

**Methods:**

The validated 26-item IKNOW-HF survey was disseminated globally to clinicians treating patients with HF. Participation was voluntary and anonymous.

**Results:**

Out of 1829 responses from 76 countries, 1285 (70.3%) were complete, predominantly from cardiologists 1031 (80.2%). Non-labelled sacubitril–valsartan dosing was reported by 1107 (86.1%) respondents, heavily driven by general cardiologists 326 (90%) and clinicians in the low- and lower-middle-income countries 195 (96%). Predictors of non-labelled dosing included practicing in Asia [odds ratio (OR) 0.347; 95% confidence interval (CI) (0.214–0.563)] and having over 10 years of experience [OR 0.695; 95% CI (0.489–0.990)]. The presence of a cardiology pharmacist trended towards a fewer non-labelled prescriptions [OR 0.542; 95% CI (0.283–1.039), *P*-value = 0.065], whereas management by HF specialist trended towards increased non-labelled usage [OR 1.443; 95% CI (0.981–2.122), *P*-value = 0.063].

**Conclusions:**

The IKNOW-HF survey reveals a substantial variation between guideline-recommended and real-world prescribing practices, with over 80% of responding clinicians utilizing non-labelled dosing. The higher prevalence among HF specialists likely reflects a pragmatic salvage strategy for advanced HF patients intolerant to standard target doses. These findings highlight the need for further education, pragmatic clinical trials evaluating real-world dosing outcomes, and broader integration of specialized pharmacists to optimize GDMT.

## Graphical Abstract

**Graphical Abstract:**
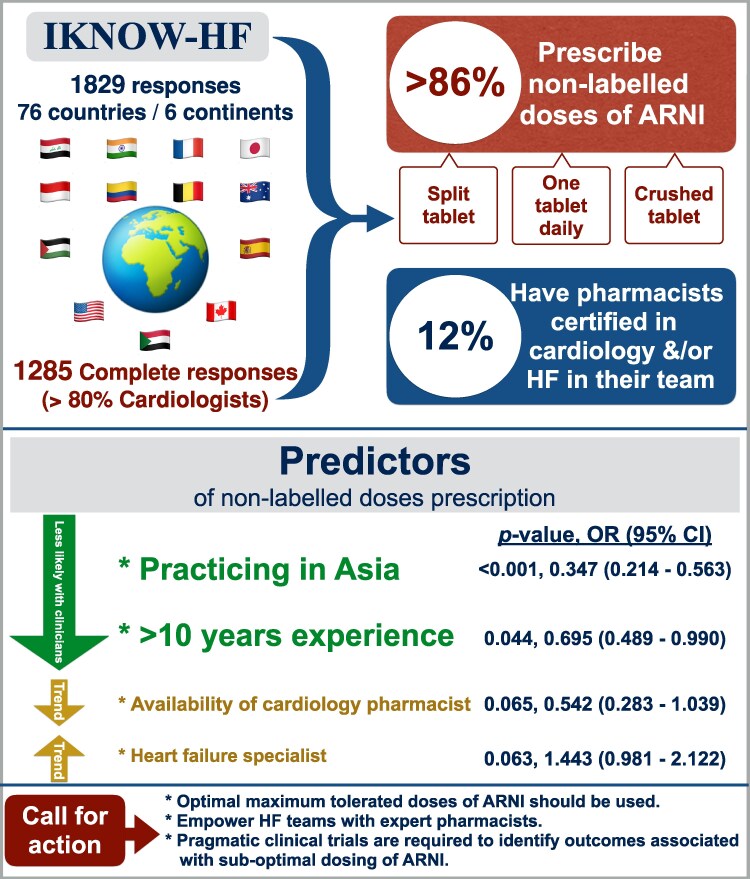
More than 1800 clinicians from around the world responded to the IKNOW-HF survey, with over 80% identifying as cardiologists. Prescribing non-labelled doses of ARNI was reported by more than 86% of respondents. These findings highlight a significant gap between evidence and practice and underscore the need for urgent action, including enhanced clinician education, greater involvement of cardiology pharmacists in HF teams, and the implementation of pragmatic clinical trials. ARNI, angiotensin receptor–neprilysin inhibitor; HF, heart failure; IKNOW-HF, International pharmacotherapy KNOWledge of Heart Failure with reduced ejection fraction.

## Introduction

Among the four foundational therapies for heart failure (HF), sacubitril–valsartan, an angiotensin receptor–neprilysin inhibitor (ARNI), is still considered a prohibitively expensive medication in most countries.^1^ At the approved maximally tolerated guideline-recommended doses for managing heart failure with reduced ejection fraction (HFrEF), sacubitril–valsartan has been shown to reduce HF hospitalizations and cardiovascular mortality by 20% versus the active comparator enalapril.^2^ The approved doses for adults with HFrEF, as per the European Medicines Agency (EMA) and US Food and Drug Administration (FDA), include 24 mg/26 mg (50 mg), 49 mg/51 mg (100 mg), and 97 mg/103 mg (200 mg) film-coated tablets, to be administered twice daily.^3,4^ Mean (±SD) dose in the PARADIGM-HF trial was 375 ± 71 mg per day.^2^ Of note, in clinical practice, a lot of patients with HF received non-target doses of angiotensin-converting enzyme inhibitors (ACEI) and angiotensin II receptor blockers (ARB).^2^ In addition, PARADIGM-HF only included patients who tolerated sacubitril–valsartan during a run-in period to ensure that ARNI could be compared with doses of enalapril previously shown to reduce mortality.^2^ Uptitration to the maximum dose of sacubitril–valsartan was only possible in up to 32% of real-world HFrEF patients in a large Belgian cohort, due to both patient characteristics as well as HF care related factors.^5^ These dosages are also endorsed by both European and American clinical guidelines.^6,7^ Phase I and II clinical trials are typically designed to determine the therapeutic dose range of new medications, balancing efficacy with safety by avoiding both toxicity at higher doses and lack of efficacy at lower doses. These pharmacological data are essential for regulatory approval by the EMA and FDA.^8^ As such, any regimen not consistent with EMA- or FDA-approved labelling; including doses below 50 mg twice daily, once-daily administration, tablet splitting, or crushing tablets to prepare capsules, should not be used, as these approaches have not been proven effective, are not supported by clinical trial evidence, and are not endorsed by current HF treatment guidelines. In addition, such practices may compromise drug absorption, alter the intended pharmacokinetic profile, and increase the risk of sub-therapeutic exposure.^3^

An online survey was conducted to evaluate the global prescribing patterns of sacubitril–valsartan, particularly the prevalence of non-labelled dosing, and the extent of involvement of cardiology pharmacists within HF teams.

## Methods

### Survey design

The *International pharmacotherapy KNOWledge of Heart Failure with reduced ejection fraction* (IKNOW-HF) project was designed as an online survey-based study, targeting over 1000 completed responses from clinicians involved in HFrEF management. Clinicians included cardiologists, physicians, pharmacists, and nurses caring for HFrEF patients. The survey comprised 26 questions: 25 required responses and one optional comment (Supplementary Table S1). The initial question addressed participant consent. The survey included multiple-choice and checkbox questions, categorized into seven domains: (i) responder demographics, (ii) guidelines and management strategies for HFrEF, (iii) sacubitril–valsartan dosing in routine practice, (iv) factors influencing ARNI prescriptions, (v) involvement of cardiology pharmacists in HF care, (vi) assessment of the knowledge of the clinicians regarding HF pharmacotherapy across different regions, and (vii) controversies in HFrEF management. The latter two domains will be reported in another publication. Pharmacists and nurses without prescribing authority were invited to respond to the survey questions regarding their clinical recommendations for physicians within a multidisciplinary team framework. Participation was voluntary and anonymous. Weekly updates on response rates were shared among investigators to encourage follow-up invitations. No financial support from industry or educational institutions was provided. The survey was administered via *SurveyMonkey* and validated in two phases: initially by 24 external experts, including HF specialists, cardiologists, pharmacists, and nurses, and subsequently by eight study co-authors. Distribution of the survey occurred through email, private messaging [WhatsApp, LinkedIn, Twitter (X)], and closed professional groups. Public sharing on social media was avoided to prevent invalid responses from non-medical personnel. The option of ‘multiple responses’ in *SurveyMonkey* was disabled to prevent multiple responses from the same respondent. The study received ethical approval from the Scientific and Research Ethical Committee of the Iraqi Board for Medical Specializations.

### Definition of non-labelled and labelled doses

Non-labelled dosing of sacubitril–valsartan was defined as any regimen deviating from EMA- or FDA-approved labelling. This included (i) low dosing (less than 50 mg twice daily), (ii) altered frequency (once-daily administration), and/or (iii) modified administration techniques (tablet splitting or crushing to prepare capsules). Conversely, the labelled dose was defined as an EMA- and FDA-recommended regimen, specifically 50 mg, 100 mg, or 200 mg tablets administered twice daily.

### Diversity information

The study leadership and authorship reflected substantial gender and geographic diversity. The principal investigator was a woman, and the two senior authors were men. The oversight committee included one man and two women. Importantly, the co-author group comprised eight women and seven men representing all six inhabited continents, including low-, middle-, and high-income countries. This broad geographic representation encompasses diverse healthcare systems, resource settings, and clinical practices in HF management, thereby ensuring the global relevance and applicability of the study findings.

### Statistical analysis

Descriptive data were expressed as numbers and percentages. Categorical variables were compared using the chi-square test. Univariate and binary multiple logistic regression analyses were performed using IBM SPSS Statistics version 26 to identify predictors of non-labelled sacubitril–valsartan prescribing. In the multiple logistic regression analysis, variables were selected based on clinical relevance and/or association in univariate analysis. Clinically important variables were retained in the multivariable model even when univariate significance thresholds were not reached, in order to avoid omission of potentially meaningful confounders. Multivariable binary logistic regression was performed using the enter method. Outcome coding: non-labelled dosing = 1, labelled dosing = 0. No missing data were identified among variables included in the regression model; therefore, all the validated responses were included in the final analysis. A two-sided *P*-value of less than 0.05 was considered statistically significant.

## Results

The survey was conducted from June 23 to 22 August 2025. Within the first 25 days, 1004 complete responses were collected. By the end of the survey period, 1829 responses from 76 countries across six continents had been obtained (Supplementary Table S2). Of these, 1285 (70.3%) participants completed all mandatory questions (*Graphical Abstract*).

### Demographics of respondents

Among the 1285 fully completed responses, 817 (63.58%) were from male participants. More than half of the respondents were based in Asia 685 (53.3%). As per the World Bank classification (Supplementary Table S3) 602 (46.8%) originated from high-income countries (HIC). The majority were physicians, 1180 (91.8%), of whom 1031 (80.23%) were cardiologists or cardiology fellows. Other physician specialties included internal medicine 135 (10.51%), general practice 27 (2.10%), and geriatrics 5 (0.39%). Full demographic details are presented in *Table 1*.

**Table 1:** Demographics of respondents

Characteristics		No. (%)
Sex	Men	817 (63.6)
	Women	468 (36.4)
Continents	Asia	685 (53.3)
	Africa	25 (1.9)
	Europe	435 (33.9)
	North America	61 (4.7)
	South America	65 (5.1)
	Australia/Oceania	14 (1.1)
Practice place	University hospital	637 (49.6)
	General/regional hospital	338 (26.3)
	Individual private practice	42 (3.3)
	Collective private practice (private hospital/clinic)	111 (8.6)
	Mixed activities	157 (12.2)
Medical specialty	Heart failure cardiologist	246 (19.1)
	Interventional cardiologist	318 (24.8)
	General cardiologist	363 (28.3)
	Heart failure fellow	18 (1.4)
	Interventional cardiology fellow	23 (1.8)
	General cardiology fellow	45 (3.5)
	Other physicians	167 (13)
	Nurse	66 (5.1)
	Clinical pharmacist	39 (3)
Years of practice in the current medical speciality (years)	1–5	477 (37)
	6–10	296 (23)
	>10	512 (40)

### Guidelines and strategies in HFrEF management

**Figure 1:**
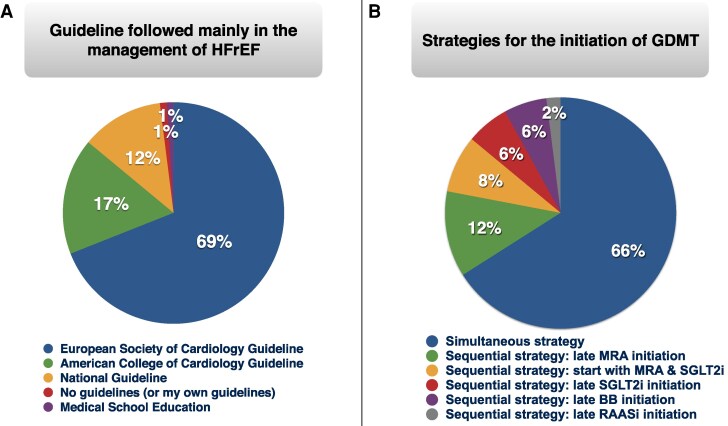
Guidelines and strategies followed for HFrEF management. (A) Guidelines for the management of HFrEF and (B) Strategies for the initiation of GDMT. BB, beta blocker; GDMT, guideline-directed medical therapy; HFrEF, heart failure with reduced ejection fraction; MRA, mineralocorticoid receptor antagonist; RAASi, renin–angiotensin–aldosterone system inhibitor; SGLT2i, sodium-glucose transport 2 inhibitor

Most respondents 882 (69%) reported the European Society of Cardiology (ESC) guidelines preference for HFrEF management. The simultaneous initiation of the four pillars of guideline-directed medical therapy (GDMT) was the predominant strategy, employed by 848 (66%) participants (*Figure 1*).

### Non-labelled dosing of sacubitril–valsartan in practice

**Figure 2:**
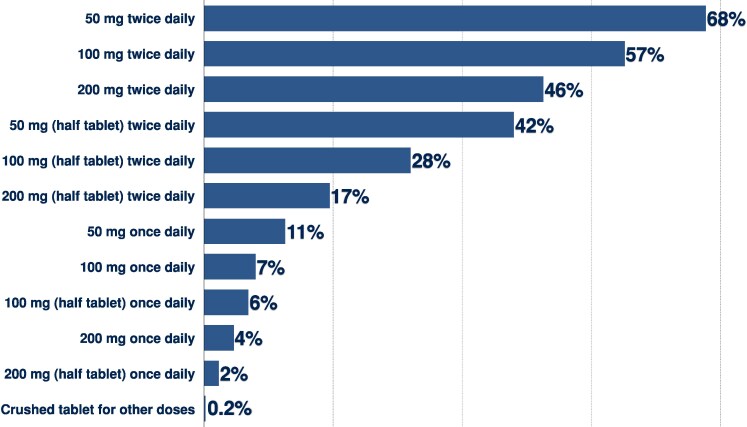
Routine dosing of sacubitril–valsartan prescribed in daily practice. Labelled and non-labelled doses of sacubitril–valsartan prescribed by clinicians in the daily clinical practice

Respondents were asked to identify all sacubitril–valsartan doses utilized in their routine clinical practice. Results showed that 50 mg tablet twice daily dosing of sacubitril–valsartan was the most frequently prescribed by respondents 874 (68%), while the non-labelled dosing of 50 mg half tablet twice daily was the fourth most frequently prescribed 545 (42%) (*Figure 2*).

**Figure 3:**
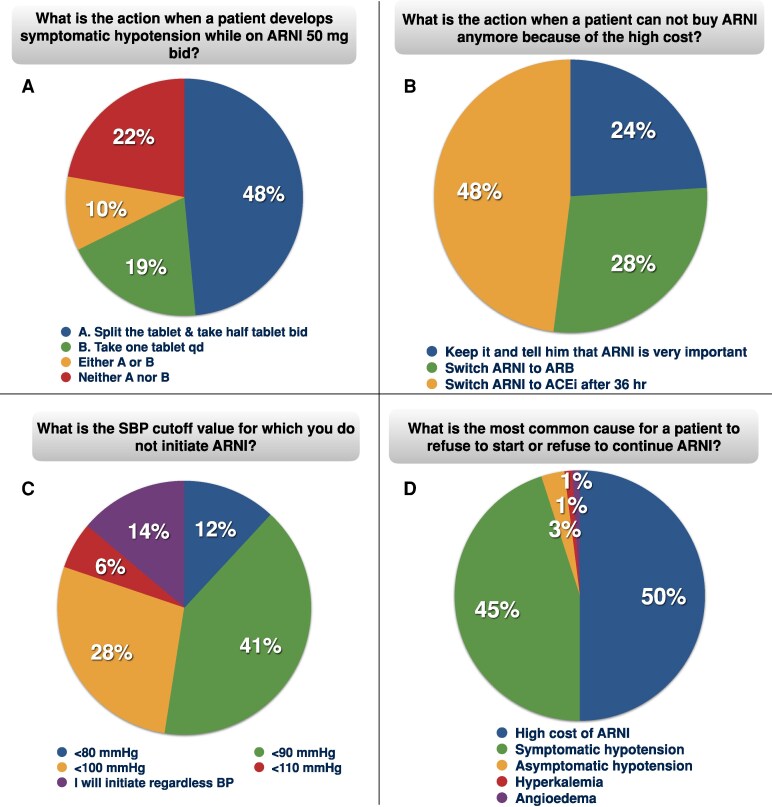
Decisions and opinions regarding sacubitril–valsartan in special clinical scenarios. (A) Dosing of sacubitril–valsartan in symptomatic hypotension. (B) Actions taken when patients cannot buy sacubitril–valsartan. (C) Systolic blood pressure cut-off value not to initiate sacubitril–valsartan. (D) Causes of patients’ refusal to take sacubitril–valsartan. ACEI, angiotensin-converting enzyme inhibitor; ARB, angiotensin receptor blocker; ARNI, angiotensin receptor–neprilysin inhibitors; bid, twice daily; BP, blood pressure; qd, Once daily; SBP, systolic blood pressure

**Figure 4:**
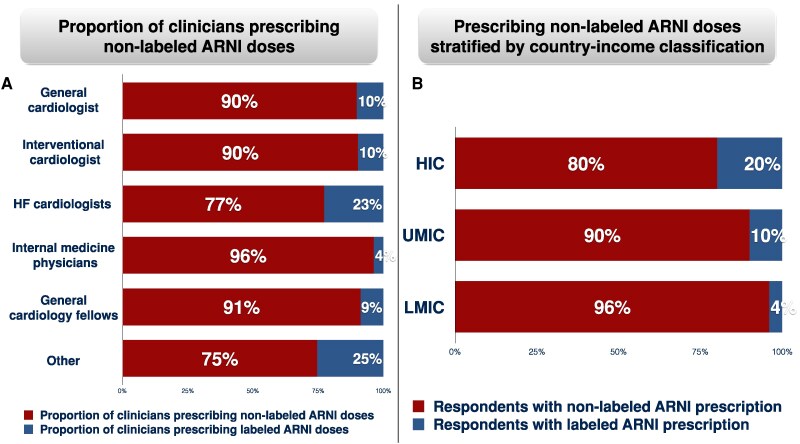
Prescription of non-labelled doses of sacubitril–valsartan. (A) Proportion of clinicians prescribing non-labelled ARNI doses stratified by medical specialty. (B) Prescribing non-labelled ARNI doses stratified by country-income classification. ARNI, angiotensin receptor–neprilysin inhibitors; HIC, high-income countries; LMIC, low- and lower-middle-income countries; UMIC, upper-middle income countries

Routine non-labelled dosing of sacubitril–valsartan was reported by 829 (64.5%) respondents. When asked about decisions in special clinical scenarios, 996 (77.5%) participants reported advising patients to split a 50 mg tablet into half and take half tablet twice daily, take the tablet once daily, or apply both approaches when symptomatic hypotension developed (*Figure 3*). *Figure 3* showed some of the reasons reported for the prescription of non-labelled doses. Combining routine and scenario-specific prescribing practices revealed that only 178 (13.85%) respondents adhered to approved therapeutic dosing, whereas 1107 (86.1%) respondents prescribed non-labelled doses in some form. Stratifying non-labelled ARNI prescriptions by specialty showed that the majority of the responding general cardiologists 326 (90%) utilized these regimens. Other healthcare professions including nurses 43 (65%), pharmacists 29 (74%), interventional cardiology fellows 19 (83%), HF fellows 14 (82%), general practitioners 23 (85%), and geriatricians 4 (80%) (*Figure 4A*). Geographically, non-labelled dosing predominated in low- and lower-middle-income countries (LMIC) 195 (96%), followed by upper-middle-income countries 431 (90%), and HICs 481 (80%), (*Figure 4B*).

### Predictors of non-labelled sacubitril–valsartan prescribing

Univariate analysis identified several significant factors influencing non-labelled prescribing, including clinician gender, continent, country income level, medical specialty, perceived drug cost (*P* < 0.001), guideline followed (*P* = 0.004), and certified cardiology pharmacist availability (*P* = 0.025) (Supplementary Tables S4 and S5).

Logistic regression indicated that clinicians practicing in Asia [odds ratio (OR) 0.347; 95% confidence interval (CI) (0.214–0.563)] and those with over 10 years of experience [OR 0.695; 95% CI (0.489–0.990)] were significantly less likely to prescribe non-labelled doses. Additionally, non-significant statistical trends were observed regarding the composition of care team: the presence of a certified cardiology pharmacist trended towards a reduction in non-labelled ARNI prescriptions [OR 0.542; 95% CI (0.283–1.039), *P*-value 0.065], whereas management by HF specialist trended towards increased non-labelled prescriptions [OR 1.443; 95% CI (0.981–2.122), *P*-value 0.063] (*Table 2*).

**Table 2:** Multivariable binary logistic regression analysis for predictors of non-labelled sacubitril–valsartan prescribing^a^

Variable	Adjusted OR (95% CI)	*P*-value
Male sex	0.851 (0.601–1.207)	0.366
Asia (vs other continents)	0.347 (0.214–0.563)	<0.001
High-income country (vs LMIC)	1.380 (0.884–2.154)	0.156
Academic practice	1.096 (0.773–1.554)	0.606
HF specialty	1.443 (0.981–2.122)	0.063
>10 years clinical experience	0.695 (0.489–0.990)	0.044
ESC guideline use	1.221 (0.772–1.930)	0.393
Simultaneous GDMT initiation	0.893 (0.621–1.284)	0.542
High ARNI cost perception	0.737 (0.513–1.059)	0.099
Certified cardiology pharmacist available	0.542 (0.283–1.039)	0.065

ARNI, angiotensin receptor–neprilysin inhibitor; ESC, European Society of Cardiology; GDMT, guideline-directed medical therapy; HF, heart failure; LMIC, low-middle-income countries.

^a^Multivariable binary logistic regression was performed using the enter method. The model demonstrated acceptable goodness-of-fit (Hosmer–Lemeshow χ^2^ = 10.125, *P* = 0.256) and was statistically significant overall (Omnibus test *P* < 0.001). The model explained 13.5% of the variance in non-labelled prescribing behaviour (Nagelkerke *R*^2^ = 0.135; Cox & Snell *R*^2^ = 0.074). Outcome coding: non-labelled dosing = 1, labelled dosing = 0. Total responses included in the analysis was 1280 after excluding 5 responses because their responses to a question about availability of pharmacists in their team were not valid.

### Involvement of cardiology pharmacists in the heart failure team

**Figure 5:**
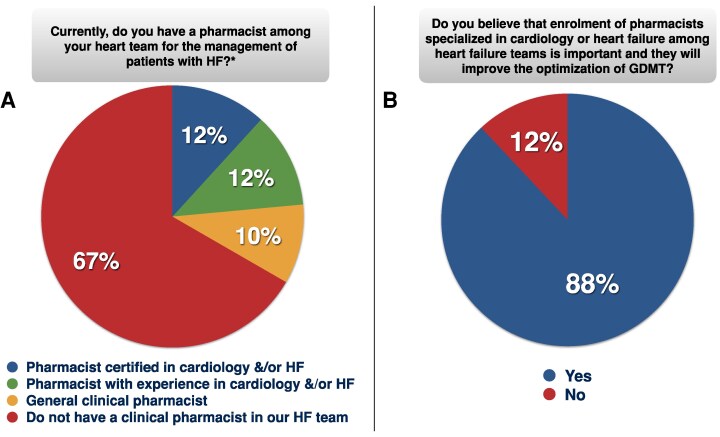
Involvement of pharmacists in the heart failure team. (A) Availability of a pharmacist in the current heart team. (B) Perceptions about the impact of the role of a specialized pharmacist in optimizing GDMT. *Among 1280 responses, 5 responses were removed because they were invalid. GDMT, guideline-directed medical therapy; HF, heart failure

Only 160 (12%) respondents reported having certified cardiology or HF pharmacists on their team. Nevertheless, 1125 (88%) expressed support for their involvement (*Figure 5*).

## Discussion

The IKNOW-HF survey highlights a global pattern of routine non-labelled dosing of sacubitril–valsartan, reported by more than four in five respondents. This practice was linked with cost and tolerability being the main determinants. Clinicians with more than 10 years of experience and those who practice in the Asian region were more likely to prescribe guideline-recommended doses. The presence of certified cardiology pharmacists was also associated with more appropriate dosing practices.

### Non-labelled dosing of sacubitril–valsartan and current evidence

The efficacy of sacubitril–valsartan in the management of HFrEF relative to ACEI/ARB has been well-established in both randomized clinical trials (RCTs) and real-world registries.^9,10^ However, there is no conclusive evidence supporting the effectiveness of non-labelled sacubitril–valsartan regimens; including doses below 50 mg twice daily, once-daily administration, tablet splitting, or the use of crushed tablets, in patients with HFrEF. Despite this lack of empirical data, some clinicians continue to prescribe these regimens based on anecdotal clinical experience, believing they offer therapeutic utility. Notably, a recent published systematic review reported an elevated risk of primary adverse outcomes associated with reduced doses of both sacubitril–valsartan and enalapril; however, these findings were derived from a post-hoc analysis of PARADIGM-HF trial.^10,11^ Two clinical studies from the USA and Korea have examined this issue; however, both included patients receiving non-labelled doses, limiting the validity of their findings.^12,13^ A recent clinical consensus statement proposed initiating sacubitril–valsartan at 25 mg twice daily in patients with asymptomatic or mildly symptomatic hypotension,^14^ but this recommendation was based on a small, single-centre observational study in which the low dose was prepared by tablet splitting, a practice that cannot ensure uniform distribution of sacubitril and valsartan.^15^ Furthermore, dose crossover occurred frequently: 21% of patients in the standard-dose group were down-titrated and 45.3% in the low-dose group required up-titration, making the absence of significant differences between groups unsurprising.^15^

### Factors affecting the non-labelled dosing of sacubitril–valsartan

Our survey identified several key factors influencing prescribing behaviour, most notably financial burden, cited by more than half of respondents. Symptomatic hypotension was the second most common concern. These barriers appear to drive clinicians towards non-labelled dosing strategies intended to reduce cost and mitigate intolerance. Notably, about half of clinicians also reported prescribing half-tablets of 100-mg or 200-mg strengths; equivalent to 50 mg or 100 mg twice daily, despite the availability of lower-dose formulations. This pattern underscores cost, rather than dose intolerance, as a major determinant of non-labelled dosing. Tablet splitting may reduce expenses but risks inaccurate dosing and raises concern that such practices may persist even after generic products become available. The aforementioned systematic review also highlighted a gap in the dosing of sacubitril–valsartan in the daily clinical practice, driven by barriers such as fear of inducing hypotension and high financial costs. Nevertheless, prescription rates for this medication have escalated five-fold over the past 7 years, fuelled by its documented clinical benefits.^10^

The PARADIGM-HF trial demonstrated a higher incidence of symptomatic hypotension with sacubitril–valsartan compared with ACEI.^2^ Nevertheless, ACEI remain alternative first-line GDMT for HFrEF.^6,7^ Accordingly, in the absence of effectiveness of non-labelled dosing of ARNI, it might be prudent to use ACEI at therapeutic doses in patients who cannot afford or tolerate sacubitril–valsartan, rather than resorting to unapproved, non-evidence-based regimens of sacubitril–valsartan that may offer limited benefit and could delay effective treatment. Additionally, one-quarter of clinicians reported continuing sacubitril–valsartan despite patients experiencing financial strain; a practice that may reduce long-term adherence in resource-limited settings. Although clinical guidelines recommend ARB strictly for ACEI-intolerant patients,^6,7^ approximately one-quarter of surveyed clinicians reported initiating ARB prior to ACEI. This finding suggests either practical clinical barriers, such as concerns regarding ACEI-induced cough, or an underlying gap in GDMT knowledge.

The trend towards increased non-labelled prescripting by HF specialists in this survey likely reflects the clinical profile of patients with advanced HF. These patients are frequently referred to tertiary specialists because they cannot tolerate even the minimum optimal target doses of an ACEI, ARB, or ARNI. Consequently, HF specialists appear to utilize non-labelled ARNI dosing as a pragmatic salvage strategy to achieve partial neurohormonal blockade, rather than due to a deficit in the clinical knowledge.

### Cardiology pharmacist involvement in the heart failure team

Gaps in pharmacotherapy knowledge likely reflect the limited integration of specialized pharmacists into HF care.^16,17^ Robust evidence indicates that cardiology-trained pharmacists improve target dosing of renin–angiotensin–aldosterone system inhibitors, including sacubitril–valsartan; yet, over two-thirds of surveyed clinicians reported no pharmacist involvement in their HF teams, despite recognizing its benefit.^7,16–18^

### GDMT initiation strategies

Encouragingly, the survey demonstrated strong momentum towards the simultaneous initiation strategy for GDMT recommended by ESC and American College of Cardiology guidelines.^7,19^ Compared with a 2024 global Heart Failure Association survey in which only 25% of respondents used a simultaneous strategy,^20^ uptake in the present survey was more than 2.5-fold higher.

In summary, the IKNOW-HF survey of over 1200 clinicians demonstrates a pervasive global reliance on non-labelled sacubitril–valsartan dosing, despite explicit guideline recommendations favouring standard therapeutic dosing. Rectifying this divergence requires multi-level interventions, including robust clinician education, targeted guideline dissemination, and the expansion of multidisciplinary HF care teams to include specialized clinical pharmacists. System-level strategies (such as electronic health record prescribing alerts and improved access to appropriate tablet strengths) are also warranted, alongside pragmatic RCTs to evaluate the clinical outcomes of modified dosing regimens. If future RCTs validate the efficacy of 25 mg tablets twice daily regimen, commercial production of this strength could mitigate the risk associated with manually splitting unscored, film-coated tablets. Splitting these formulations can result in unequal doses and disrupt the functional film coating, risking sub-optimal drug delivery, altered pharmacokinetics, and subsequently clinical worsening. Ultimately, for patients who cannot tolerate or afford sacubitril–valsartan, reverting to ACEI or ARB (when ACEI are contraindicated) remains the guideline-supported standard of care, particularly with the resource-limited settings.

### Strengths and limitations of the study

The IKNOW-HF was an international large registry to assess the issue of non-labelled dosing of ARNI, involving a diverse sample of multidisciplinary clinicians, with global perspective. Also, non-labelled dosing phenomenon was quantified and defined, key factors were identified, regional variability and practice were assessed, pharmacist involvement were evaluated, and actionable recommendations were provided. Nevertheless, several limitations must be considered when interpreting these findings. First, the deployment of convenience and snowball sampling methodologies via digital platforms (e.g. WhatsApp, LinkedIn, X) may introduce selection bias, potentially over-representing clinicians who are highly active on social media or within specific professional networks. To mitigate this risk, we selectively targeted closed professional groups and issued direct invitations to senior clinicians to capture a broad spectrum of clinical experience. Second, although the survey captured data across 76 countries, the high proportion of respondents from Asia (53.3%) may limit the generalizability of these findings to regions with vastly different healthcare infrastructures and resource allocations. Third, the survey did not directly assess clinicians’ knowledge of EMA- and FDA-approved therapeutic doses of sacubitril–valsartan. Therefore, the observed rate of non-labelled dosing should not be interpreted solely as a knowledge deficit regarding approved dosing. Fourth, we did not record the total number of medical personnel who received the survey invitation. Therefore, we were unable to calculate the survey response rate or evaluate the potential impact of non-response bias.

Another major limitation is the lack of information about patients’ outcomes, and therefore no inferences can be made on whether non-labelled doses influence or not HFrEF patients’ morbidity and mortality. Finally, not all confounding factors may have been adjusted for in the multivariable analysis. These important limitations do not negate the strengths of our survey which included nearly 2000 responders across six continents and included countries across the income spectrum.

## Conclusions

The IKNOW-HF survey data highlights a substantial variation between guideline-recommended and real-world sacubitril–valsartan prescribing patterns, with over 80% of responding clinicians reporting non-labelled dosing regimens. Optimizing alignment with evidence-based pharmacotherapy warrants further exploration into clinician education, pragmatic trials assessing real-world dosing strategy outcomes, and the potential supportive role of specialized cardiology pharmacists.

## Supplementary Material

xvag162_Supplementary_Data

## Declarations

### Data Availability

The data underlying this article will be shared on reasonable request to the corresponding author.

### Pre-registered Clinical Trial Number

None supplied.

## Appendix 1 On behalf of the IKNOW-HF investigators group:

**1. Oversight committee**

Alexander Lyon (United Kingdom), Karen Sliwa (South Africa), Dania Mohty (Saudi Arabia).

**2. Investigators**

**Austria:** Peter P. Rainer. **Azerbaijan:** Yasmin Rustamova. **Bulgaria:** Mariya Tokmakova. **Denmark:** Anders Barasa, Julie KK Vishram-Nielsen. **Germany:** Amr Abdin. **Greece:** Peggy Kostakou. **Indonesia:** Novi Yanti Sari. **Iraq:** Zainab Mustafa Mahdi. **Poland:** Marta Zaleska Kociecka. **Qatar:** Rasha Kaddoura. **Romania:** Laura Antohi. **Saudi Arabia:** Dania Mohty, Mirvat Alasnag. **Serbia:** Marija Zdravkovic, Goran Loncar, Ivan Milinković. **Slovenia:** Anja Zupan Mežnar. **South Africa:** Karen Sliwa. **Sweden:** Carmen Basic. **Switzerland:** David Niederseer, Federica Guidetti. **United Kingdom:** Alexander Lyon, Priya Reehal. **United States of America:** Ilya Giverts, Oumol Khairy El Moctar El Houssein.
