# Supplementary material for: Global trend and predictors of non-labelled sacubitril–valsartan dosing: results from IKNOW-HF survey
Source: ESC Heart Fail. 2026 Jun 6;13(3):xvag162. doi: 10.1093/eschf/xvag162 (PMC13282903; doi:10.1093/eschf/xvag162)
Supplement: xvag162_Supplementary_Data [file xvag162_supplementary_data.docx]

**Supplemental Tables**

| **Supplemental Table 1**: Survey Questionnaire |
| --- |
| Q1: Do you agree to participate in this study, anonymously and voluntarily? Acknowledging that you are a physician, pharmacist, or nurse, you will answer this survey only once, and your answers will serve in a global analysis and scientific publications, and you will not share the link of the survey publicly in social medias?   - Yes - No |
| Q2: What is your sex? |
| Q3: What is your country of current practice? |
| Q4: What is your type of practice ? (single answer allowed)   - University Hospital - General/regional hospital - Individual Private practice - Collective Private practice (private hospital/clinic) - Mixed activities |
| Q5: What is your medical specialty? (single answer allowed)   - Heart failure cardiologist - Interventional cardiologist - General cardiologist - General practitioner - Internal medicine physician - Geriatrician - Heart Failure Cardiology -Fellow in training (FIT) - Interventional Cardiology FIT - General cardiology FIT - Cardiology pharmacist but I’m not authorized to prescribe guideline-directed medical therapy (GDMT) - Cardiology pharmacist and I’m authorized to prescribe GDMT - Clinical pharmacist but I’m not authorized to prescribe GDMT - Clinical pharmacist and I’m authorized to prescribe GDMT - Nurse but I’m not authorized to prescribe GDMT - Nurse and I’m authorized to prescribe GDMT |
| Q6: For how many years have you been practicing in the current medical specialty? (Type a whole number only) |
| Q7: Which one of the following clinical guidelines do you depend on mainly while treating patients with HFrEF? (single response allowed)   - European Society of Cardiology Guidelines - American College of Cardiology Guidelines - National Guidelines - Medical School Education - No guidelines (or my own guidelines) |
| Q8: Which one of the following strategies do you mainly follow while treating newly diagnosed patients with HFrEF, euvolemic, with no comorbid disease? (assume the patient has a heart rate of 75 bpm, blood pressure 130/70 mmHg, and eGFR=70 ml/min, K=4,0 mmol/l). (single response allowed)   1. Simultaneous strategy: starting beta blockers, RAASi, SGLT2i, and MRA all at the same time 2. Sequential strategy: with beta blocker to be added at the last step 3. Sequential strategy: with RAASi to be added at the last step 4. Sequential strategy: with SGLT2i to be added at the last step 5. Sequential strategy: with MRA to be added at the last step 6. Sequential strategy: start with MRA and SGLT2i to potentialize diuretic efficiency and reduce the risk of hypokalemia. |
| Q9: If the only available RAASi medications are ACEI and ARB, which one will you initiate in a patient newly diagnosed with HFrEF with no comorbid disease? (single response allowed)   - ACEI - ARB |
| Q10: A patient with HFrEF with no comorbid disease, was initiated on sacubitril/valsartan as initial treatment. However, 2 months later he informs you he can not buy sacubitril/valsartan because of the high cost. What is your action plan based on your daily practice? (single response allowed)   - Keep him on sacubitril/valsartan and tell him that this medication is very important and better than ACEI and ARB - Switch sacubitril/valsartan to ARB - Switch sacubitril/valsartan to ACEI after 36 hours - Sacubitril/valsartan is not available in our country |
| Q11: Please select all the doses of sacubitril/valsartan that you prescribe in the daily clinical practice for patients with HFrEF. (Multiple responses allowed). (Note: dose of 50 mg = 24 mg/26 mg, 100 mg = 49 mg/51 mg, 200 mg = 97 mg /103 mg )   - 50 mg (half tablet) once daily - 50 mg (half tablet) twice daily - 50 mg (tablet) once daily - 50 mg (tablet) twice daily - 100 mg (half tablet) once daily - 100 mg (half tablet) twice daily - 100 mg (tablet) once daily - 100 mg (tablet) twice daily - 200 mg (half tablet) once daily - 200 mg (half tablet) twice daily - 200 mg (tablet) once daily - 200 mg (tablet) twice daily - Crush sacubitril/valsartan tablet to prepare capsule with individualized doses per patient - Sacubitril/valsartan is not available in our country |
| Q12: For patients with HFrEF and symptomatic hypotension on sacubitril/valsartan 50 mg tablet twice daily, what is your advice to the patient? (single response allowed). (Note: dose of 50 mg = 24 mg/26 mg)   - Split the tablet into two halves and take half tablet twice daily - Take one tablet once daily - Either first or second option - Neither first nor second option - Sacubitril/valsartan is not available in our country |
| Q13: According to your clinical experience, what is the most common cause for a patient to refuse to start or refuse to continue administration of sacubitril/valsartan? (single response allowed)   - High cost of sacubitril/valsartan - Symptomatic hypotension - Asymptomatic hypotension - Hyperkalemia - Angioedema - Sacubitril/valsartan is not available in our country |
| Q14: In a patient with newly diagnosed HFrEF, what is the systolic blood pressure value for which you do not initiate sacubitril/valsartan? (single response allowed)   - <80 mmHg - <90 mmHg - <100 mmHg - <110 mmHg - I will initiate regardless the blood pressure as long as the patient has asymptomatic hypotension - Sacubitril/valsartan is not available in our country |
| Q15: Which one of the following GDMT should not be prescribed in a patient with prostate cancer on abiraterone who developed cancer therapy-related cardiac dysfunction (CTRCD) with LVEF <40%? (single response allowed)   - Lisinopril - Carvedilol - Empagliflozin - Spironolactone - Ivabradine - I don’t know |
| Q16: For a patient with a history of HFrEF and currently has improved LVEF > 50% and normal NTproBNPs, what do you change or not in your prescription? (single response allowed)   - Keep the same quadruple therapy (Beta blocker, RAASi, MRA, SGLT2i) - Stop all quadruple therapies - Stop beta blocker - Stop RAASi - Stop MRA - Stop SGLT2i |
| Q17: For a patient with a history of HFrEF who underwent cardiac resynchronization therapy (CRT) implantation and now his LVEF > 50%, do you keep him on: (single response allowed)   - Keep the same quadruple therapy - Stop all quadruple therapies - Stop beta blockers - Stop RAASi - Stop MRA - Stop SGLT2i |
| Q18: For patients with HFrEF with LVEF less than 30%, do you prescribe oral anticoagulants for ambulatory patients? (single response allowed)   - Yes, prophylactic dose of oral anticoagulant - Yes, therapeutic dose of oral anticoagulant - No, as long as there is no other indication |
| Q19: Do you prescribe nebivolol as a GDMT beta blocker for patients with HFrEF? (single response allowed)   - Yes, because it is recommended by ESC guidelines - Yes, because it is recommended by our national guidelines - No, because it is not mentioned among GDMT beta blockers in ACC guidelines - No, because I believe the current available studies don’t show survival benefit for patients with HFrEF - No, because it is not recommended by our national guidelines |
| Q20: What is the starting dose of sacubitril-valsartan in patients with moderate hepatic impairment (Child-Pugh B, bilirubin 4 mg/dL, prothrombin time 8 second, with moderate ascites)? (single response allowed). (Note: dose of 50 mg = 24 mg/26 mg, 100 mg = 49 mg/51 mg)   - 50 mg once daily - 50 mg twice daily - 100 mg twice daily - I don’t know |
| Q21: For patients with HFrEF planning to undergo bariatric surgery, should SGLT2i be stopped pre-operative? (single response allowed)   - Yes, 1 days before surgery - Yes, 2 days before surgery - Yes, 3 days before surgery - No, no need to be stopped - I don’t know |
| Q22: Which one of the following GDMT should be taken with food? (single response allowed)   - Ramipril - Metoprolol - Spironolactone - Dapagliflozin - Ivabradine - I don’t know |
| Q23: Do you prescribe sacubitril/valsartan for patients with HFrEF to be crushed and prepared in capsules with doses other than the doses of (24/26 mg, 49/51 mg, 97/103 mg)? (single response allowed)   - Yes - No - Sacubitril/valsartan is not available in our country |
| Q24: Do you believe that enrollment of pharmacists specialized in cardiology or heart failure among heart failure teams is important and they will improve the optimization of GDMT?   - Yes - No |
| Q25: Currently, do you have a pharmacist among your heart team for the management of patients with HF? (multiple response allowed)   - Yes, we have a pharmacist certified in cardiology and/or heart failure - Yes, we have a pharmacist with experience in cardiology and/or heart failure - Yes, we have a clinical pharmacist but neither certified nor expertise in cardiology and/or heart failure - No, we do not have a clinical pharmacist among our heart failure team |
| Q26: Do you have any additional comment? (Optional) |

| **Supplemental Table 2: Seventy six countries participated in the IKNOW-HF survey** | | | | | |
| --- | --- | --- | --- | --- | --- |
| **Country** | **No. (%)** | **Country** | **No. (%)** | **Country** | **No. (%)** |
| Iraq | 272 (16.61) | Brunei | 6 (0.37) | Rwanda | 2 (0.12) |
| India | 203 (12.39) | Greece | 6 (0.37) | Slovenia | 2 (0.12) |
| France | 164 (10.01) | Pakistan | 6 (0.37) | Sweden | 2 (0.12) |
| Japan | 119 (7.26) | United Arab Emirates | 6 (0.37) | Afghanistan | 1 (0.06) |
| Indonesia | 94 (5.74) | Germany | 5 (0.31) | Algeria | 1 (0.06) |
| Poland | 93 (5.68) | Netherlands | 5 (0.31) | Bangladesh | 1 (0.06) |
| Azerbaijan | 85 (5.19) | Malaysia | 4 (0.24) | Bosnia and Herzegovina | 1 (0.06) |
| Colombia | 85 (5.19) | Senegal | 4 (0.24) |  |  |
| Spain | 70 (4.27) | South Africa | 4 (0.24) | Burkina Faso | 1 (0.06) |
| Belgium | 59 (3.60) | Sudan | 4 (0.24) | Cameroon | 1 (0.06) |
| Serbia | 46 (2.81) | Austria | 3 (0.18) | Cyprus | 1 (0.06) |
| United States of America | 40 (2.44) | Brazil | 3 (0.18) | Djibouti | 1 (0.06) |
| Saudi Arabia | 29 (1.77) | Dominican Republic | 3 (0.18) | Ghana | 1 (0.06) |
| Canada | 25 (1.53) | Mexico | 3 (0.18) | Iran | 1 (0.06) |
| Turkey | 21 (1.28) | Palestine | 3 (0.18) | Kuwait | 1 (0.06) |
| Russia | 16 (0.98) | Portugal | 3 (0.18) | Lebanon | 1 (0.06) |
| Australia | 15 (0.92) | Singapore | 3 (0.18) | Libya | 1 (0.06) |
| Italy | 15 (0.92) | Chile | 2 (0.12) | Madagascar | 1 (0.06) |
| United Kingdom | 14 (0.85) | Egypt | 2 (0.12) | Mozambique | 1 (0.06) |
| Kenya | 10 (0.61) | Jordan | 2 (0.12) | Nigeria | 1 (0.06) |
| Switzerland | 10 (0.61) | Moldova | 2 (0.12) | Oman | 1 (0.06) |
| Argentina | 8 (0.49) | Montenegro | 2 (0.12) | Philippines | 1 (0.06) |
| Denmark | 8 (0.49) | Morocco | 2 (0.12) | Qatar | 1 (0.06) |
| Bulgaria | 7 (0.43) | Niger | 2 (0.12) | Syria | 1 (0.06) |
| South Korea | 7 (0.43) | North Macedonia | 2 (0.12) | Tunisia | 1 (0.06) |
| Tanzania | 7 (0.43) | Romania | 2 (0.12) |  |  |

** 191 of 1829 respondents skipped the survey before responding to a question of country of work.*

| **Supplemental Table 3: Participated countries based on World Bank classification*** | | |
| --- | --- | --- |
| **HIC** | **UMIC** | **LMIC** |
| Australia, Austria, Belgium, Brunei, Bulgaria, Canada, Chile, Denmark, France, Germany, Greece, Italy, Japan, Korea Republic, Kuwait, Netherlands, Oman, Poland, Portugal, Qatar, Romania, Russian Federation, Saudi Arabia, Singapore, Slovenia, Spain, Sweden, Switzerland, United Arab Emirate, United Kingdom, and United States of America | Argentina, Azerbaijan, Bosnia and Herzegovina, Brazil, Colombia, Dominican Republic, Indonesia, Iran, Iraq, Libya, Malaysia, Mexico, Moldova, Montenegro, North Macedonia, Serbia, South Africa, and Turkey | Afghanistan, Egypt, Ghana, India, Jordan, Kenya, Lebanon, Madagascar, Morocco, Mozambique, Nigeria, Pakistan, Palestine, Rwanda, Senegal, Sudan, Syria, Tanzania, and Tunisia |

* For 1285 participants with completed responses to the survey.

*HIC= high income countries; UMIC= upper middle income countries; LMIC= low and lower middle income countries.*

| **Supplemental Table 4:** Univariate analysis of participants baseline characteristics between labeled versus non-labeled prescription of sacubitril-valsartan. | | | | | |
| --- | --- | --- | --- | --- | --- |
| **Variables** | | **Total**  **(1285)** | **labeled**  **(178)** | **Non-labeled**  **(1,107)** | **p-value** |
| Sex | Man | 817 (63.6) | 92 (7.2) | 725 (56.4) | **< 0.001** |
|  | Woman | 468 (36.4) | 86 (6.7) | 382 (29.7) |  |
| Continents | Asia | 685 (53.3) | 42 (3.3) | 643 (50) | **< 0.001** |
|  | Africa | 25 (1.9) | 6 (0.5) | 19 (1.5) |  |
|  | Europe | 435 (33.9) | 112 (8.7) | 323 (25.1) |  |
|  | North America | 61 (4.7) | 11 (0.9) | 50 (3.9) |  |
|  | South America | 65 (5.1) | 5 (0.4) | 60 (4.7) |  |
|  | Australia/ Oceanian | 14 (1.1) | 2 (0.2) | 12 (0.9) |  |
| Country- income level | HIC | 602 (46.8) | 121 (9.4) | 481 (37.4) | **< 0.001** |
|  | UMIC | 479 (37.3) | 48 (3.7) | 431 (33.5) |  |
|  | LMIC | 204 (15.9) | 9 (0.7) | 195 (15.2) |  |
| Practice field | University hospital | 637 (49.6) | 104 (8.1) | 533 (41.5) | 0.066 |
|  | General/ regional hospital | 338 (26.3) | 40 (3.1) | 298 (23.2) |  |
|  | Individual private practice | 42 (3.3) | 2 (0.2) | 40 (3.1) |  |
|  | Collective private hospital/ clinic | 111 (8.6) | 11 (0.9) | 100 (7.8) |  |
|  | Mixed activity | 157 (12.2) | 21 (1.6) | 136 (10.6) |  |
| Career years | 1 - 5 year | 477 (37.1) | 70 (5.4) | 407 (31.7) | 0.234 |
|  | 6 - 10 year | 296 (23) | 47 (3.7) | 249 (19.4) |  |
|  | 10 year or more | 512 (39.8) | 61 (4.7) | 451 (35.1) |  |

Data represented as No. (%)

*HIC= high income countries; UMIC= upper middle income countries; LMIC= low and lower middle income countries.*

| **Supplemental Table 5:** Univariate analysis for labeled versus non-labeled doses prescription of sacubitril-valsartan. | | | | | |
| --- | --- | --- | --- | --- | --- |
| **Variables** | | **Total**  **(1285)** | **labeled**  **(178)** | **Non-labeled**  **(1,107)** | **p-value** |
| Medical specialty | HF cardiologist | 264 (20.5) | 59 (4.6) | 205 (16) | **< 0.001** |
|  | Interventional cardiologist | 341 (26.5) | 35 (2.7) | 306 (23.8) |  |
|  | General cardiologists | 408 (31.8) | 41 (3.2) | 367 (28.6) |  |
|  | Other physicians | 167 (13) | 10 (0.8) | 157 (12.2) |  |
|  | Pharmacist | 39 (3) | 10 (0.8) | 29 (2.3) |  |
|  | Nurse | 66 (5.1) | 23 (1.8) | 43 (3.3) |  |
| Guideline | ESC | 882 (68.6) | 144 (11.2) | 738 (57.4) | **0.004** |
|  | ACC | 219 (17) | 18 (1.4) | 201 (15.6) |  |
|  | National | 155 (12.1) | 15 (1.2) | 140 (10.9) |  |
|  | Medical school | 10 (0.8) | 0 (0) | 10 (0.8) |  |
|  | No guideline | 19 (1.5) | 1 (0.1) | 18 (1.4) |  |
| Strategy | Sequential | 437 (34) | 63 (4.9) | 374 (29.1) | 0.674 |
|  | Simultaneous | 848 (66) | 115 (8.9) | 733 (57) |  |
| Cost of ARNI | No | 637 (49.6) | 114 (8.9) | 523 (40.7) | **< 0.001** |
|  | Yes | 648 (50.4) | 64 (5) | 584 (45.4) |  |
| Certified cardiology pharmacist availability* | No | 1125 (87.5) | 165 (12.8) | 960 (74.7) | **0.025** |
|  | Yes | 160 (12.5) | 13 (1) | 147 (11.4) |  |

** for 1280 respondents.*

*Data represented as No. (%)*

*ACC= American College of Cardiology; ARNI=* angiotensin receptor-neprilysin inhibitor*; ESC= European Society of Cardiology; HF= heart failure.*
